# Association between Skin Cancer and Systemic and Ocular Comorbidities in South Korea

**DOI:** 10.3390/jcm10112451

**Published:** 2021-06-01

**Authors:** Sul Hee Lee, Jun-Soo Ro, Kee Yang Chung, Sang Hoon Lee, Young Lip Park, Jung Eun Kim, Si Hyung Lee

**Affiliations:** 1 Department of Dermatology, Soonchunhyang University Bucheon Hospital, Soonchunhyang University College of Medicine, 170 Jomaru-ro, Bucheon 14584, Korea; dermalsh@schmc.ac.kr (S.H.L.); shlee@schmc.ac.kr (S.H.L.); ylpark@schmc.ac.kr (Y.L.P.); 2 Department of Health Policy and Management, Seoul National University College of Medicine, 103 Daehak-ro, Jongno-gu, Seoul 03080, Korea; geniuses1@snu.ac.kr; 3 Department of Dermatology, Cutaneous Biology Research Institute, Yonsei University College of Medicine, 50 Yonsei-ro, Seodaemun-gu, Seoul 03722, Korea; kychung@yuhs.ac; 4 Department of Dermatology, Soonchunhyang University Cheonan Hospital, Soonchunhyang University College of Medicine, 31 Suncheonhyang 6-gil, Dongnam-gu, Cheonan 31151, Korea; 96839@schmc.ac.kr; 5 Department of Ophthalmology, Soonchunhyang University Hospital Bucheon, Soonchunhyang University College of Medicine, 170 Jomaru-ro, Bucheon 14584, Korea

**Keywords:** skin cancer, systemic comorbidities, ocular comorbidities

## Abstract

Background: In this study, we investigated the associations between various systemic and ocular comorbidities and skin cancer, in a nationwide cohort of South Koreans. Method: We reviewed the data of 1,103,302 individuals in the South Korean National Health Insurance Service-National Sample Cohort database from 2002 to 2015. Of these, 1202 individuals diagnosed with skin cancer from 2004 were included in the study group. The control group was matched in a 1:5 ratio based on propensity scores. Results: The prevalence rates of melanoma and non-melanoma skin cancer increased from 2004 to 2015. Multivariate logistic regression analysis revealed that, among the various systemic conditions, hypertension was significantly associated with skin cancer, while among ocular comorbidities, macular degeneration showed a significant association with skin cancer. Conclusions: This is the first study to demonstrate associations between skin cancer and various systemic and ocular comorbidities. The results suggest that hypertension and macular degeneration may increase the risk of skin cancer development, or vice versa. Further studies are needed to evaluate the causal relationships between these conditions.

## 1. Introduction

Skin cancer is a common malignancy in Western countries. Although the risk factors and comorbidities of skin cancer have been investigated worldwide, they remain controversial due to the nature and complex pathogenesis of cancer. Various conditions including hypertension [1], diabetes mellitus (DM) [2], Parkinson’s disease [3], atopic dermatitis [4], and vitiligo [5] have been suggested as possible comorbidities of skin cancer, but the relationship between skin cancer and other diseases remains unclear. The eye is another structure commonly exposed to sunlight, and previous studies have indicated a possible association of skin cancer and ocular conditions including cataract [6], and pterygium [7,8]. However, despite the fact that the skin and eyes are the two organs frequently exposed to the sun, thus sharing ultraviolet (UV) radiation as a risk factor for various skin and ocular diseases, investigations of various ocular comorbidities of skin cancer-based on large samples have rarely been performed. Therefore, there is a need to investigate the association between skin cancer, systemic comorbidities, and ocular conditions to evaluate possible risk factors.

In Asian countries, skin cancer occurs less frequently than in Western countries. Epidemiologic studies have reported that the prevalence and annual incidence of skin cancer in Asian countries vary greatly according to the country where the study was conducted [9,10,11]. Ethnic differences, regional differences in access to care, and discordant study design may also affect the wide range of reported estimates. In South Korea, however, both melanoma and non-melanoma skin cancer (NMSC) have been steadily increasing [10]. As this raises concerns about possible comorbidities of skin cancer in the Korean population, we investigated the prevalence of skin cancer from 2004 to 2015 in South Korea. Based on data taken from a nationwide cohort that included a large population sample, we also investigated the association between skin cancer and various cardiovascular comorbidities including DM, hypertension, hyperlipidemia, coronary artery disease, myocardial infarction, stroke, and other conditions such as chronic obstructive pulmonary disease (COPD), Parkinson’s disease and irritable bowel syndrome, which have previously been reported to be associated with skin cancer. In addition, the association between skin cancer and several ocular diseases affected by sun exposure, including pterygium, cataract, and macular degeneration was also investigated. 

## 2. Materials and Methods

### 2.1. Database Used in the Study

In South Korea, the National Health Insurance Service (NHIS) is a mandatory single medical insurer system, in which all citizens in South Korea are obligated to enroll. The NHIS first developed the NHIS-National Sample Cohort (NSC) for research purposes, including the data of 1,025,340 individuals from 2002 to 2013. Recently, NHIS-NSC 2002–2015 was released including 1,103,302 selected population follow-up data from 2002 to 2015. In this study, we used data from the NHIS-NSC 2004–2015 for the analysis. The design and protocol of this study were approved by the Institutional Review Board of Yonsei University Severance Hospital (approval number 4-2019-0387). This study was conducted in accordance with the principles of the Declaration of Helsinki, and written informed consent was not required for this study.

### 2.2. Selection of Study Samples

Skin cancer cases were defined as those who had the Korean Classification of Diseases (KCD) codes C43 and C44 as the main diagnosis. Patients diagnosed with skin cancer from 2004 to 2015 were included in the analysis. As a result, 1202 skin cancer cases were included.

Control subjects without skin cancer diagnosis codes were selected using propensity scores with nearest-neighbor matching, with a 5:1 ratio to skin cancer patients. Ultimately, 6010 control subjects were included. Propensity scores were calculated using sociodemographic parameters, including age group (<50, 50–59, 60–69, 70–79, ≥80 years), sex, income (≤40th, 41st–70th, ≥71st percentiles), residential area (city, rural residents), and Charlson comorbidity index (CCI; score < 3, ≥3), to adjust for the frequency of hospital visits.

### 2.3. Prevalence and Comorbidities

To determine the prevalence of skin cancer, participants diagnosed with skin cancer who visited a dermatologist at least twice from 2004 to 2015 were considered. The annual prevalence was calculated based on the total number of cases per year and the total population qualified for NHIS-NSC on 1 January of each enrollment year.

Systemic and ocular comorbid condition codes included in the analysis were DM (E10–E14), hypertension (I10–I15), hyperlipidemia (E78.0–E78.5), pterygium (H110), cataract (H25), macular degeneration (H35.30, H35.34, H35.39), actinic keratosis (L570), Parkinson’s disease (G20), coronary artery disease (I20, I25), myocardial infarction (I21–I24), inflammatory bowel disease (IBD; K50–K52), stroke (I64), and COPD (J44), diagnosed between 2002 and 2008. Since the purpose of this study was to investigate cross-sectional associations between systemic or ocular diseases and skin cancer, we did not consider the date when the diagnosis was made during the study period.

### 2.4. Statistical Analyses

The baseline characteristics are shown as a number and percentage, and a chi-square test was used to compare the groups. Multivariate logistic regression analysis was performed to calculate the odds ratio (OR), with a 95% confidence interval (CI), to evaluate the potential associations between skin cancer and various systemic and ocular diseases. Variables included in the multivariate analyses were age, sex, income, residential area, CCI, and comorbidities previously reported to be associated with skin cancer. A two-sided *p*-value less than 0.05 was considered statistically significant. All statistical analyses were conducted using SAS software, version 9.4 (SAS Institute, Cary, NC, USA) and R 3.4.1 (R Project for Statistical Computing, Vienna, Austria).

## 3. Results

**Figure 1:**
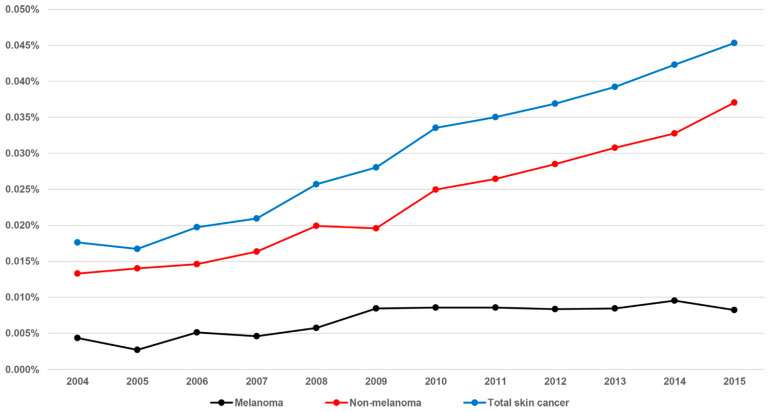
Prevalence of melanoma, non-melanoma skin cancer, and total skin cancer during the study period.

The prevalence of skin cancer during the 12-year period is shown in Table 1 and Figure 1. The prevalence rates of total skin cancer per 10,000 persons during the study period were 1.8, 1.7, 2.0, 2.1, 2.6, 2.8, 3.4, 3.5, 3.7, 3.9, 4.2, and 4.5, in 2004, 2005, 2006, 2007, 2008, 2009, 2010, 2011, 2012, 2013, 2014, and 2015, respectively, showing an increasing trend. When divided into two subgroups, NMSC and melanoma, a similar increasing trend in the prevalence of NMSC was observed from 2004 (1.3 per 10,000 persons) to 2015 (3.7 per 10,000 persons), while the trend in the prevalence of melanoma showed a plateau pattern throughout the same study period (0.4 per 10,000 persons in 2004, and 0.8 per 10,000 persons in 2015).

Table 2 shows the baseline characteristics of the study population. A total of 1202 skin cancer patients and 6010 controls were included for comparison. There were no significant differences in age group, sex, income, residential area, and CCI, the variables used for propensity score matching (*p* = 1.000). Among various systemic comorbidities reported to be associated with skin cancer, a significantly higher prevalence of hypertension (*p* = 0.001), hyperlipidemia (*p* = 0.049), and coronary artery disease (*p* = 0.019) was detected in skin cancer subjects compared to the controls. Among ocular diseases previously reported to be related to sun exposure, including cataract, macular degeneration, and pterygium, only macular degeneration showed a higher prevalence in the skin cancer group than the controls (*p* = 0.019).

Multivariate logistic regression analysis showed that participants aged >80 years showed a significant association with lower odds of skin cancer compared to those aged <50 years (OR: 0.676; 95% CI: 0.519–0.881; *p* = 0.006), after adjusting for confounding factors (Table 3). Among various systemic comorbidities, skin cancer was significantly associated only with hypertension (*p* = 0.001), while hyperlipidemia and coronary artery disease did not show a significant association. The analysis also showed that skin cancer was significantly associated with macular degeneration (OR: 1.500; 95% CI: 1.080–2.082; *p* = 0.016).

## 4. Discussion

The incidence of NMSC and of cutaneous melanoma has been increasing in South Korea for several years, together with nationwide awareness and interest in the two diseases in the Korean population. Little information was available previously because few studies have been performed regarding the trends in skin cancer, due to its low incidence and prevalence in Asian countries. The age-standardized incidence rates of cutaneous melanoma, squamous cell carcinoma (SCC), and basal cell carcinoma during 1999–2014 in South Korea were reported to be 0.66, 1.34, and 2.45 per 100,000 men and 0.58, 1.04, and 2.07 per 100,000 women, respectively [10]. The incidence rate of skin cancer in Singapore was reported to be 7.4 per 100,000 person-years during 2003–2006 [9]. In this study, we observed that the overall prevalence of skin cancer in South Korea was <0.05% every year, but steadily increasing during 2004–2015. During the study period, although the prevalence rate of melanoma and melanoma in situ changed after 2009, with a doubling of the rate in 2015 compared to 2004, the prevalence of NMSC has been increasing steadily; almost three times higher than melanoma and melanoma in situ each year. Although these rates are much lower than those of a previously reported study based on a Western population [12], the increase in the prevalence of skin cancer in South Korea is a public health concern, as the importance of early detection and prevention of skin cancer should be emphasized.

Although no significant association between DM and skin cancer was observed in this study, the association between the two diseases, and particularly with diabetic drugs, has been widely investigated; however, the reported results have been controversial. According to a large sample size study from China, the risk of skin cancer was increased in male patients with type 2 DM [13]. However, regarding anti-diabetic drugs, metformin had been suggested to lower skin cancer, especially NMSC risk in a dose-response pattern based on a nationwide study from Taiwan [2]. Another study suggested that Taiwanese patients with type 2 DM who had used rosiglitazone for >13 months, or with a cumulative dose >1750 mg, have a lower risk of NMSC [14]. Due to these drug effects, the complex association between skin cancer and DM should be further evaluated in future studies.

In this study, unlike DM, hypertension showed a significant association with skin cancer after adjusting for potential confounding factors. Hypertension is one of the most common systemic diseases worldwide and has been suggested to be a comorbidity associated with various cancers. Regarding skin cancer, antihypertensive drugs, rather than hypertension itself, have been suggested to have a role in developing skin cancer. The photosensitizing nature of some antihypertensive drugs such as α-2 receptor agonists and diuretics were reported to act as a carcinogen when exposed to UV radiation, thus increasing the risk of developing skin cancer [15,16,17]. Moreover, a significant association with NMSC and melanoma was also observed for calcium channel blockers and β-blockers in a meta-analysis study from Italy [1]. Another study showed that long-term use of angiotensin II receptor blockers was associated with a risk of malignant melanoma, and long-term diuretic use was associated with a risk of SCC [15]. On the other hand, a conflicting result was also reported from Korea that the risk of developing melanoma was significantly lower in patients taking hydrochlorothiazide, which suggested that the anti-cancer activity of hydrochlorothiazide may have overwhelmed the carcinogenetic effect induced by photosensitivity of the drug [18]. While the relationship between some specific malignancies such as renal cell carcinoma [19,20], breast cancer [21], and hypertension has been investigated, the mechanism of the association between skin cancer and hypertension itself has not been proven. In addition to photosensitive antihypertensive drugs, the Wnt and AMPK pathway-associated endothelial dysfunction observed in both cancer and chronic hypertension may also contribute to the association between the two diseases [22]. In addition, although small numbers of patients with skin cancer receive chemotherapy, Alkylating agents, such as cisplatin and carboplatin, used to treat advanced cutaneous SCC have been reported to increase the risk of hypertension [23,24].

In this study, we investigated the relationship between skin cancer and several ocular diseases including cataract, pterygium, and macular degeneration. Both human skin and eyes are susceptible to UV radiation. In addition to photoaging and neo-melanogenesis, carcinogenesis is one of the main reactions of the skin to chronic UV damage. Regarding the eyes, acute exposure of the eyes to UV radiation increases the risks of photokeratitis and photoconjunctivitis [25]. However, chronic exposure to UV light is closely related to solar retinopathy, pterygium, cataract, and SCC of the cornea and conjunctiva [25].

Due to limited previous studies, the association between ocular diseases and skin cancer remains obscure. In a few studies, pterygium has been reported to have an association with skin cancer. A cohort study from Western Australia demonstrated an association between pterygium and melanoma and suggested that pterygium was an indicator of increased risk of cutaneous melanoma [8]. Another population-based study from Taiwan demonstrated that the risk of NMSC was increased in patients with pterygium [7]. Unfortunately, in our study, the association between skin cancer and pterygium was not significant. We assume that demographic risk factors related to pterygium, such as occupational history, residence, alcohol consumption, and smoking history, which we could not obtain from the provided cohort data [26], may have affected the results.

Previous studies investigating the association between skin cancer and cataract, one of the most common ocular diseases worldwide, have shown discordant results. Although UV radiation is considered an important risk factor, cataract has a complex pathogenesis and includes risk factors such as age, genetic factors, smoking, systemic comorbidities including diabetes and hypertension, and corticosteroid use [27]. Exposure to UV radiation from sunlight has been considered as a risk factor for both skin cancer and cataract. A recent cross-sectional study from Australia demonstrated that the prevalence of skin cancer was higher in patients with cataracts than in those without [6]. However, there is also conflicting evidence of sunlight exposure as a possible risk factor for cataracts. A population-based study from Korea analyzing the risk factors of all subtypes of cataracts reported no significant association between a longer duration of sun exposure and all subtypes of cataracts after adjusting for confounding factors [28]. Our study confirmed this result, showing no significant association between cataract and skin cancer in the Korean population. Ethnic variations and the complex pathogenesis of cataract development may have led to the disparity between our results and those of previous studies.

In this study, a significant association between macular degeneration and skin cancer was observed, which has not been reported previously. Macular degeneration is a leading cause of irreversible blindness for those aged >50 years in developed countries [29]. Although not clearly defined, long-term accumulation of solar radiation has been suggested as an important risk factor, causing photochemical damage of the retina and inducing macular degeneration [30], together with age, smoking, and various systemic conditions such as DM. Skin cancer also shares a similar pathogenetic mechanism, with UV radiation being its most important risk factor of tumorigenesis [31,32], which may explain the significant association between the two diseases found in our study.

Additionally, we also looked for any association between skin cancer and Parkinson’s disease or IBD, which have been suggested as possible comorbidities associated with skin cancer in several previous studies. The association between melanoma and Parkinson’s disease has been demonstrated in several previous studies [3,33]. In addition to L-dopa treatment in patients with Parkinson’s disease, several common genes found in both patients with Parkinson’s disease and melanoma have been suggested to be involved in the association between these two diseases [3,33]. Recently, in addition to melanoma, an increased risk of developing basal cell carcinoma has also been reported in patients with Parkinson’s disease [3]. However, in this study, a significant association between Parkinson’s disease and skin cancer was not observed. The much lower incidence and prevalence of cutaneous melanoma in the Korean population compared to Western populations may contribute to the non-significant results found in this study. Regarding IBD, a moderately increased risk of NMSC in patients with IBD using thiopurines has been previously demonstrated [34,35]. However, in this study, the association between IBD and skin cancer was also not significant. Further investigations are needed to clarify the association of Parkinson’s disease and IBD with skin cancer.

Some limitations should be considered. First, because of the cross-sectional nature of the study, causal relationships between skin cancer and the various comorbidities could not be defined. Second, although UV radiation is considered the most important overall risk factor of skin cancer, the commonest subtype of melanoma skin cancer in Asian countries, unlike most Western countries, is acral lentiginous melanoma (ALM), which usually develops in sun-protected areas, and is unrelated to UV radiation [36]. ALM is mainly associated with a predisposing genetic defect and old age, and is less commonly associated with nevi and prior sunburns [37]. Due to the unique etiology of ALM, the results of this study may not explain its complex association with various conditions. However, several suggested comorbidities that showed statistical significance in this study share UV radiation as possible related factors, demonstrating their relationship. Considering the approximately three to four times lower prevalence of overall melanoma compared to NMSC in the Korean population, we assume that the etiology of NMSC, rather than melanoma, had a far greater influence on the results of this study, demonstrating the association between the systemic comorbidities investigated and skin cancer. Third, due to the overall extremely low prevalence of skin cancer, we could not perform subgroup analyses to investigate the association between NMSC or melanoma and the various systemic comorbidities. In addition, our study was entirely based on the KCD code system, and therefore, information on the subtypes of either melanoma skin cancer or NMSC could not be obtained. Thus, we were unable to evaluate the specific associations between the systemic comorbidities investigated and each subtype of skin cancer.

Nonetheless, this study had several strengths. First, it was based on data taken from a nationwide survey that included a large population sample, and selection bias may have been minimized. Second, we demonstrated the prevalence rates of melanoma and NMSC in South Korea, which were not widely known. Third, to the best of our knowledge, this is the first nationwide population-based cohort study to investigate the association between skin cancer and various ocular diseases in Asian countries. Importantly, this is also the first study to demonstrate comprehensively the association between skin cancer and various systemic and ocular comorbidities. Based on the results of this study, dermatologists should pay more attention to the signs and symptoms of hypertension or macular degeneration when examining skin cancer patients. Moreover, patients with hypertension or macular degeneration should be more alert to any signs of skin cancer.

Given the few Asian population-based studies investigating the association between skin cancer and other possible comorbidities, our study confirms and expands previous findings regarding the associated systemic and ocular diseases of skin cancers in an Asian population.

## Figures and Tables

**Table 1:** Estimated prevalence rates of total skin cancer, NMSC, and melanoma (per 10,000 persons) during the study period.

Year	2004	2005	2006	2007	2008	2009	2010	2011	2012	2013	2014	2015
Population	804,790	848,240	876,546	892,730	902,580	923,373	828,801	933,289	943,205	945,707	954,779	957,535
Total skin cancer												
Prevalence	142	142	173	187	232	259	278	327	348	371	404	434
Per 10,000 persons	1.8	1.7	2.0	2.1	2.6	2.8	3.4	3.5	3.7	3.9	4.2	4.5
NMSC												
Prevalence	107	119	128	146	180	181	207	247	269	291	313	355
Per 10,000 persons	1.3	1.4	1.5	1.6	2.0	2.0	2.5	2.6	2.9	3.1	3.3	3.7
Melanoma												
Prevalence	35	23	45	41	52	78	71	80	79	80	91	79
Per 10,000 persons	0.4	0.3	0.5	0.5	0.6	0.8	0.9	0.9	0.8	0.8	1.0	0.8

NMSC, non-melanoma skin cancer.

**Table 2:** Demographic characteristics of the population included in the study.

	Total *n* (%)	Cancer *n* (%)	No Cancer *n* (%)	*p*-Value
Age				1.000
<50	1032	172 (16.7)	860 (83.3)	
50–59	1014	169 (16.7)	845 (83.3)	
60–69	1350	225 (16.7)	1125 (83.3)	
70–79	1872	312 (16.7)	1560 (83.3)	
>80	1944	324 (16.7)	1620 (83.3)	
Sex				1.000
Male	3252	542 (16.7)	2710 (83.3)	
Female	3960	660 (16.7)	3300 (83.3)	
Income				1.000
70–100 percentile (low)	3804	634 (16.7)	3170 (83.3)	
40–70 percentile	1740	290 (16.7)	1450 (83.3)	
>40 percentile (high)	1668	278 (16.7)	1390 (83.3)	
Residential area				1.000
City resident	2994	499 (16.7)	2495 (83.3)	
Rural resident	4218	703 (16.7)	3515 (83.3)	
CCI				1.000
<3	4044	674 (16.7)	3370 (83.3)	
≥3	3168	528 (16.7)	2640 (83.3)	
Diabetes mellitus				0.965
Yes	2824	470 (16.6)	2354 (83.4)	
No	4388	732 (16.7)	3656 (83.3)	
Hypertension				0.001 *
Yes	4288	765 (17.8)	3523 (82.2)	
No	2924	437 (14.9)	2487 (85.1)	
Hyperlipidemia				0.049 *
Yes	3402	598 (17.6)	2804 (82.4)	
No	3810	604 (15.9)	3206 (84.1)	
Parkinson’s disease				0.686
Yes	151	27 (17.9)	124 (82.1)	
No	7061	1175 (16.6)	5886 (83.4)	
Coronary artery disease				0.019 *
Yes	259	57 (22.0)	202 (78.0)	
No	6953	1145 (16.5)	5808 (83.5)	
Myocardial infarction				0.828
Yes	156	27 (17.3)	129 (82.7)	
No	7056	1175 (16.7)	5881 (83.3)	
Inflammatory bowel disease				0.788
Yes	2843	478 (16.8)	2365 (83.2)	
No	4369	724 (16.6)	3645 (83.4)	
Stroke				0.408
Yes	107	21 (19.6)	86 (80.4)	
No	7105	1181 (16.6)	5924 (83.4)	
COPD				0.284
Yes	506	93 (18.4)	413 (81.6)	
No	6706	1109 (16.5)	5597 (83.5)	
Cataract				0.153
Yes	2927	510 (17.4)	2417 (82.6)	
No	4285	692 (16.1)	3593 (83.9)	
Macular degeneration				0.019 *
Yes	233	52 (22.3)	181 (77.7)	
No	6979	1150 (16.5)	5829 (83.5)	
Pterygium				0.131
Yes	343	47 (13.7)	296 (86.3)	
No	6869	1155 (16.8)	5714 (83.2)	

CCI: Charlson comorbidity index; COPD: chronic obstructive pulmonary disease. * Statistically significant (*p* < 0.05).

**Table 3:** Multivariable logistic regression analysis evaluating the association between various systemic and ocular comorbidities and skin cancer.

	OR (95% CI)	*p*-Value
Age		
<50	Ref.	
50–59	0.896 (0.703–1.140)	0.302
60–69	0.842 (0.662–1.072)	0.772
70–79	0.754 (0.586–0.971)	0.171
>80	0.676 (0.519–0.881)	0.006 *
Sex		
Male	1.025 (0.899–1.169)	0.713
Female	Ref.	
Income		
70–100 percentile (low)	Ref.	
40–70 percentile	1.009 (0.862–1.180)	0.942
>40 percentile (high)	1.006 (0.857–1.181)	0.983
City		
City resident	1.020 (0.896–1.161)	0.761
Rural resident	Ref.	
CCI		
<3	Ref.	
≥3	0.968 (0.724–1.293)	0.824
Diabetes mellitus		
Yes	0.923 (0.686–1.242)	0.597
No	Ref.	
Hypertension		
Yes	1.380 (1.111–1.539)	0.001 *
No	Ref.	
Hyperlipidemia		
Yes	1.060 (0.920–1.221)	0.422
No	Ref.	
Parkinson’s disease		
Yes	1.026 (0.660–1.593)	0.910
No	Ref.	
Coronary artery disease		
Yes	1.320 (0.956–1.821)	0.091
No	Ref.	
Myocardial infarction		
Yes	0.926 (0.594–1.442)	0.733
No	Ref.	
Irritable bowel disease		
Yes	0.967 (0.848–1.102)	0.614
No	Ref.	
Stroke		
Yes	1.158 (0.689–1.946)	0.580
No	Ref.	
COPD		
Yes	1.101 (0.858–1.412)	0.449
No	Ref.	
Cataract		
Yes	1.055 (0.899–1.238)	0.515
No	Ref.	
Macular degeneration		
Yes	1.500 (1.080–2.082)	0.016 *
No	Ref.	
Pterygium		
Yes	0.789 (0.571–1.091)	0.152
No	Ref.	

CCI: Charlson comorbidity index; COPD: chronic obstructive pulmonary disease; OR: odds ratio; CI: confidence interval; Ref.: reference. * Statistically significant (*p* < 0.05).

## Data Availability

The data collected and analyzed in the study will be available from the corresponding author on reasonable request.
